# Otolaryngologists, Pediatricians, and Emergency and Family Medicine Physicians Adherence to Acute Otitis Media Diagnosis and Management Guidelines: A Retrospective Study in a Saudi Arabian Tertiary Center

**DOI:** 10.7759/cureus.18492

**Published:** 2021-10-05

**Authors:** Abdulaziz S Alhammad, Salman AlOtieschan, Abdulrahman Alsalim, Eyad Alshehri, Mohammed Al-Kadi, Jihad Nassar

**Affiliations:** 1 Radiology, College of Medicine, King Saud University, Riyadh, SAU; 2 Medicine, College of Medicine, King Saud Bin Abdulaziz University for Health Sciences, Riyadh, SAU; 3 Radiology, King Faisal Specialist Hospital and Research Centre, Riyadh, SAU; 4 Otolaryngology - Head and Neck Surgery, King Abdulaziz Medical City, Riyadh, SAU

**Keywords:** pediatrics, acute otitis media, antibiotic, otitis media, saudi arabia

## Abstract

Background

Acute otitis media (AOM) is one of the most common conditions in the pediatric population and a common reason for physicians to prescribe antibiotics. Most children will develop otitis media at least once during their life.

Objectives

Our study aimed to evaluate and compare the adherence of family medicine physicians, otolaryngologists, pediatricians, and emergency medicine physicians to the American Academy of Pediatrics and American Academy of Family Physicians guidelines for the diagnosis and the management of AOM.

Methods

This is a retrospective study that was conducted at the Ministry of National Guard - Health Affairs affiliated hospitals and primary clinics in Riyadh, Saudi Arabia. All patients diagnosed with AOM and treated between 2016 and 2019 were included in the study. Exclusion criteria included any patient above the age of 18 years old or patients with incomplete data on their files. Variables included demographic data of the patients and treating physicians, associated infections, and whether the treating physician followed the diagnosis and management guidelines.

Results

Most patients were below the age of two years. Emergency medicine physicians were the most common to treat patients with AOM. Although most documentations were sufficient, 39.8% were insufficient or not written. Most (74%) physicians adhered to the diagnosis guidelines, while 57.5% adhered to management guidelines.

Conclusion

Although most physicians adhered to the diagnosis and treatment guidelines, stressing on the matter is essential to avoid unnecessary antibiotics use. We recommend further prospective studies with a bigger sample size of more than one center to have a more accurate reflection of the current situation.

## Introduction

Otitis media (OM) is a group of middle ear conditions and includes acute otitis media (AOM) and otitis media with effusion (OME); it is commonly seen as a pathological condition in children and adolescents [1]. AOM is defined as an acute inflammation of the middle ear space characterized by otalgia, fever, irritability, and poor feeding in young children [2]. On the other hand, OME is the presence of free fluid in the middle ear without any symptoms of acute infections [1]. OME is most commonly preceded by AOM, but it can also occur independently [2]. In the United States, around 60-80% of children develop AOM within their first year of life, while 80-90% will develop AOM between two and three years of age [2]. The peak incidence of AOM usually occurs between six and 24 months of age [2]. AOM is the most common cause of outpatient clinic visits in the United States and the United Kingdom [2]. In the United States, a national survey found that 30% of all antibiotics prescribed to patients younger than 18 years of age were to treat AOM [2]. In the United Kingdom, AOM is the most common childhood infection, and antibiotics were prescribed in 87% of total AOM cases treated by primary health care clinics [2].

Etiological factors for AOM include Eustachian tube dysfunction secondary to an upper respiratory tract infection (URTI), increased negative pressure in the middle ear, accumulation of middle ear fluid, and microbial growth [2]. The most common organisms that cause AOM are strains of *Streptococcus* pneumonia not in the heptavalent pneumococcal conjugate vaccine (PCV7), non-typeable *Haemophilus influenzae* (NTHi), and *Moraxella catarrhalis* [1-3]. Daycare attendance and younger ages are acknowledged as risk factors for developing AOM among children [2]. Other risk factors include using a pacifier, being a male, white ethnicity, history of enlarged adenoids tonsillitis or asthma, and history of ear infection in parents or siblings [3].

The American Academy of Pediatrics (AAP) and the American Academy of Family Physicians (AAFP) developed guidelines for the diagnosis and management of AOM [1,2]. Previously, the diagnosis criteria were based on symptomatology without any evidence of inflammation on the otoscope. The updated criteria endorse the ear examination by using an otoscope for diagnosis in conjunction with clinical symptoms [2]. AOM diagnosis requires acute onset of otorrhea accompanied by moderate-to-severe tympanic membrane bulging, not caused by otitis externa, or mild bulging of the tympanic membrane with acute ear pain [2]. Initial treatment is recommended for symptoms including otalgia, fever, and irritability [1,3]. For children older than six months with otorrhea or severe signs or symptoms (moderate or severe otalgia, otalgia for at least 48 hours, or temperature of 39°C or higher), antibiotic therapy is recommended for 10 days [2]. For children older than six months and less than two years old with bilateral AOM, without severe signs or symptoms, antibiotic therapy is recommended for 10 days [2]. For children older than six months and less than two years old, observation or antibiotic therapy is recommended for 10 days [2]. For children older than two years without severe signs or symptoms, observation or antibiotic therapy is recommended for five to seven days [2]. In OME, antibiotics are not recommended for treatment [3,4]. It is important for physicians to diagnose and manage AOM properly. OME is often misdiagnosed with AOM, leading to overtreatment and unnecessary prescription of antibiotics, which increases the risks of antibiotic resistance and the emergence of superbugs [2,3].

In Saudi Arabia, there has not been sufficient data collection for the prevalence of AOM and OME despite the high number of cases presented in daily practice. It is also worth noting that King Abdulaziz Medical City (KAMC) does not have any implemented guidelines for the diagnosis and management of AOM. The significance of this research is to assess and compare the adherence of otolaryngologists, pediatricians, and emergency and family medicine physicians to the AAP and AAFP guidelines for the diagnosis and management of AOM. In this research, we assess the procedure and protocols used by physicians from different specialties in confirming the diagnosis as well as the management in terms of antibiotic choice, dosage, and treatment duration. This study will shed more light on the current practice used in the diagnosis and management of AOM and will help us to come up with recommendations to improve the current practice for the diagnosis and management of AOM among the pediatric population.

## Materials and methods

This is a retrospective study conducted at the Ministry of National Guard - Health Affairs (MNG-HA) affiliated hospitals and primary clinics in Riyadh, Saudi Arabia. All patients who were diagnosed with AOM and treated in MNG-HA hospitals or primary clinics between 2016 and 2019 were included in the study. We designed our data collection sheet, and reviewed and collected data from patients' charts using the BESTCare system (Electronic Medical Record). Each patient was anonymously coded by assigning them a unique serial number. The data collection sheet contains the demographic data of the patients, such as age and gender, the type of clinic they have visited, their doctor's specialty, and their doctor's level of experience. In addition to the type of AOM and the description of examination and associated infections, we also assessed physician’s adherence to the AAP and AAFP guidelines for the diagnosis and management of AOM, as well as physicians’ adherence to correct antibiotic dosage according to the AAP and AAFP guidelines.

Definitions

Acute otitis media: Acute inflammation of the middle ear space characterized by otalgia, fever, irritability, and poor feeding in young children.

Sufficient description of examination: Full description of the tympanic membrane appearance bilaterally.

Insufficient description of examination: The appearance of the tympanic membrane not fully described, the tympanic membrane not visualized, or no description was written in patient records.

Ethical considerations 

The study was reviewed and approved by an Institutional Review Board Committee at King Abdullah International Medical Research Centre (#RC19/141/R). All data were collected by the authors and kept confidential in a safe place.

Statistical analysis

Data were checked for completeness and correctness. Demographic data, clinic and physicians’ data, and AOM-related data were presented as frequencies and percentages. Associations between variables of interest were observed using the chi-square test. The analysis was performed in a 95% confidence interval using the Statistical Package for Social Science (SPSS), version 24.0 (IBM, Armonk, NY, USA).

## Results

A total of 924 cases were enrolled in this study, of which 52.8% were males. The age group of 25-60 months was the most common (28.6%) followed by the age group of 7-12 months (27.5%) and the age group of 13-24 months (21.8%). Least common were patients under the age of six months (11.4%) and patients above the age of 60 months (10.8%). Patients mainly presented with unilateral AOM without otorrhea, representing 36.1% of our patients. Moreover, 32.5% of the patients presented with otorrhea with AOM followed by unilateral or bilateral AOM with severe symptoms (22.6%). Lastly, bilateral AOM without otorrhea represented 8.8% of the patients.

Most of the patients with AOM were seen by emergency medicine physicians (90.6%) followed by otolaryngologists (6.2%), pediatricians (2.8%), and family medicine physicians (0.4%). Regarding the level of experience, most were staff physicians (55.8%) followed by residents (27.9%), assistant consultants (6.3%), consultants (5%), and associate consultants (3.8%), and least common were fellows (1.2%).

Most documentations were found to be sufficient (60.2%), while 31.7% were insufficient and 8.1% were not written. The majority (66%) of the patients did not have a documented recurrent episode, while 16.1% had one recurrent episode, 7.6% had two recurrent episodes, 4.7% had three recurrent episodes, and 5.6% had more than three recurrent episodes. Those who didn’t have a follow-up visit documented represented 77.3%, while 12.7% had one follow-up visit, 4.5% had two follow-up visits, 2.5% had three follow-up visits, and 3% had four or more follow-up visits. Most patients did not have any associated infection (61.8%), while 33.3% of patients had a URTI, 2.5% had laryngitis, 1% had gastroenteritis, 0.6% had urinary tract infection, 0.4% had bronchiolitis, and 0.2% had conjunctivitis.

In regard to adherence to the guidelines, 74% and 57.7% of the physicians adhered to the diagnosis and treatment guidelines, respectively. When stratified based on the age group, 53.3% of physicians adhered to the treatment guidelines when encountering patients younger than six months. Moreover, 63% and 51.9% of physicians adhered to the treatment guidelines when treating patients in the age group of 6-24 months and those older than 24 months old, respectively. Most physicians who followed the diagnosis guidelines were emergency medicine physicians (90.5%) followed by otolaryngologists (6.4%), pediatricians (2.8%), and family medicine physicians (0.3%). Similarly, most physicians who followed the treatment guidelines were emergency medicine physicians (88.3%) followed by otolaryngologists (7.9%), pediatricians (3.4%), and family medicine physicians (0.4%). However, the association between the doctor’s specialty and adherence to the diagnosis guidelines was not statistically significant (p-value of 0.713). While the association between the doctor’s specialty and adherence to the treatment guidelines was statistically significant (p-value of 0.33). We also found that 36.1% of cases where treatment guidelines were not followed had an associated infection. However, the association was not statistically significant (p-value of 0.256). Moreover, the majority (66.2%) of physicians who adhered to the treatment guidelines also adhered to the diagnosis guidelines. Physicians who did not adhere to the treatment guidelines mostly did not adhere to the diagnosis guidelines too (67.5%). The association between following the diagnosis and treatment guidelines was statistically significant (p-value of <0.001).

As shown in Table 1, only 8.8% of patients with associated infections received the correct dose of antibiotic, while 91.2% of patients either received a higher or lower dose than needed. Moreover, the association between receiving an incorrect dose of antibiotic and having an associated infection was statistically significant (p-value of <0.001). Of all physicians, 20.7% did not prescribe an antibiotic. As for physicians who prescribed an antibiotic, 38.5% of them gave the correct dose. On the other hand, 39% prescribed a lower dose than the patient’s need according to his/her weight and 1.7% gave a higher dose.

**Table 1:** Antibiotics prescription and associated infections *Includes both not prescribing any antibiotic or not prescribing the recommended antibiotic.

Variable	Associated infection	
	Yes (n = 353)	No (n = 571)
Did the treating physician prescribe an antibiotic according to the guidelines?		
No*	0 (0)	192 (33.6)
Yes, correct dose	31 (8.8)	325 (56.9)
Yes, overdosed	13 (3.7)	3 (0.5)
Yes, underdosed	309 (87.5)	51 (8.9)

## Discussion

AOM is one of the most common conditions in the pediatric population and a common reason for physicians to prescribe antibiotics. Approximately 80% of all pediatric population will suffer from otitis media during their lifetime [4]. Our objective in this study was to assess physicians’ adherence to AAFP/AAP treatment and diagnosis guidelines.

As we have mentioned earlier, KAMC has not yet implemented any guidelines to diagnose and manage AOM. This might be one of the attributing elements to the lack of adherence to AAFP/AAP guidelines. Another element that might reflect the lack of adherence to the guidelines is incomplete documentation of the history and examination, preventing us from knowing whether the physician followed the guidelines or not.

In a meta-analysis conducted by Bourgeois et al., after compiling 19 articles looking at adherence to current AAP/AAFP guidelines for the diagnosis and management of AOM, they identified major barriers that contributed to some degree in poor adherence to the guidelines [5]. Those barriers were due to physicians’ lack of knowledge on the current AAFP/AAP guidelines [5]. Another factor that may play a role is doubts regarding follow-up, which lead to more cautious therapeutic and diagnostic plans. In a study that found around 20% of antibiotics were wrongly prescribed, 74% of patients were not examined by a physician prior to the emergency department visit [6]. This may explain why most of the patients were treated inappropriately.

McCormick et al. concluded that physician’s poor adherence to guidelines was attributed to parents’ poor knowledge regarding the benefits and harms of using antibiotic and the general course of the disease [7]. This has increased the pressure on physicians to prescribe antibiotics even though the patient might have not met the criteria [7]. In addition, parents became overwhelmed with their children’s symptoms, falsely believing that antibiotic will relieve their symptoms, which solidifies the misconception [7].

AOM is found to be slightly more common in males than females, which matches our results as we found that the percentage of males is slightly higher than females [8,9]. Similar to what we found in our study, highest rate of incidence was found to be in children up to the age of four years [8,9]. AOM is considered one of the most common causes to visit the clinic [10]. In this study, most patients were diagnosed and treated at the emergency department, which may be due to the young age of patients and coexistent infections such as URTI.

Identifying the symptoms of AOM can be difficult, as one of the best indicators is otalgia. It can be challenging to diagnose AOM in many children, as they would present with non-specific signs and symptoms such as ear pulling, irritability, and poor feeding, which brings us to the diagnosis guidelines as it previously depended on symptomatology, but that is not the case anymore. It requires objective findings (e.g., bulging of tympanic membrane and otorrhea) to minimize inaccurate diagnoses and results in unnecessary antibiotic prescription. We found that most patients presented with either bilateral or unilateral signs of AOM without otorrhea. Similar to previous studies, it was found that the most common sign or symptom was redness of the tympanic membrane [11,12]. However, our study has presented higher rates of otorrhea compared to previous studies [12,13], which may be an indication of late presentation of our patients, resulting in delayed initiation of treatment. A recent meta-analysis concluded that treatment with antibiotics reduces the rate of perforation and otorrhea of AOM by 50% [14].

We recommend further prospective studies to assess the physicians’ adherence to diagnosis and treatment guidelines and factors affecting their adherence to diagnosis and treatment guidelines. Moreover, it may be essential to raise public awareness about when to visit the emergency department in order to avoid unnecessary visits.

Although we included all patients diagnosed with AOM, poor documentation and using different diagnosis codes may affect the number of subjects included in our study.

## Conclusions

In conclusion, although no current guidelines for the diagnosis and treatment of AOM are implemented at KAMC, we found that most physicians followed the diagnosis and treatment AAP/AAFP guidelines. However, a handful of physicians were not following the treatment guidelines, which may contribute to bacterial resistance to antibiotics. Most patients were seen by emergency medicine physicians, which may be attributed to the severity of symptoms and/or young age of the patients. There was an association between prescribing incorrect low doses of antibiotics and concurrent infections in patients. Further prospective studies are recommended to accurately measure the adherence to diagnosis and treatment guidelines as well as their implications on the patients' outcome.
